# Mitochondrial DNA Released by Trauma Induces Neutrophil Extracellular Traps

**DOI:** 10.1371/journal.pone.0120549

**Published:** 2015-03-16

**Authors:** Kiyoshi Itagaki, Elzbieta Kaczmarek, Yen Ting Lee, I. Tien Tang, Burak Isal, Yashar Adibnia, Nicola Sandler, Melissa J. Grimm, Brahm H. Segal, Leo E. Otterbein, Carl J. Hauser

**Affiliations:** 1 Department of Surgery, Beth Israel Deaconess Medical Center/Harvard Medical School, Boston, MA, United States of America; 2 Center for Vascular Biology Research, Beth Israel Deaconess Medical Center/Harvard Medical School, Boston, MA, United States of America; 3 Departments of Medicine and Immunology, Roswell Park Cancer Institute, Buffalo, NY, United States of America; 4 Department of Medicine, University at Buffalo School of Medicine, Buffalo, NY, United States of America; University of Tokyo, JAPAN

## Abstract

Neutrophil extracellular traps (NETs) are critical for anti-bacterial activity of the innate immune system. We have previously shown that mitochondrial damage-associated molecular patterns (mtDAMPs), including mitochondrial DNA (mtDNA), are released into the circulation after injury. We therefore questioned whether mtDNA is involved in trauma-induced NET formation. Treatment of human polymorphoneutrophils (PMN) with mtDNA induced robust NET formation, though in contrast to phorbol myristate acetate (PMA) stimulation, no NADPH-oxidase involvement was required. Moreover, formation of mtDNA-induced NETs was completely blocked by TLR9 antagonist, ODN-TTAGGG. Knowing that infective outcomes of trauma in elderly people are more severe than in young people, we measured plasma mtDNA and NET formation in elderly and young trauma patients and control subjects. MtDNA levels were significantly higher in the plasma of elderly trauma patients than young patients, despite lower injury severity scores in the elderly group. NETs were not visible in circulating PMN isolated from either young or old control subjects. NETs were however, detected in PMN isolated from young trauma patients and to a lesser extent from elderly patients. Stimulation by PMA induced widespread NET formation in PMN from both young volunteers and young trauma patients. NET response to PMA was much less pronounced in both elderly volunteers’ PMN and in trauma patients’ PMN. We conclude that mtDNA is a potent inducer of NETs that activates PMN via TLR9 without NADPH-oxidase involvement. We suggest that decreased NET formation in the elderly regardless of higher mtDNA levels in their plasma may result from decreased levels of TLR9 and/or other molecules, such as neutrophil elastase and myeloperoxidase that are involved in NET generation. Further study of the links between circulating mtDNA and NET formation may elucidate the mechanisms of trauma-related organ failure as well as the greater susceptibility to secondary infection in elderly trauma patients.

## Introduction

Since its initial description by Brinkmann [1], the formation of NETs has been widely studied [2, 3], and found to be a fundamental mechanism of pathogen surveillance and killing by PMN. After activation by stimulants including microbes, PMN undergo a distinct form of programmed cell death associated with chromatin decondensation and a release of extracellular DNA filaments covered with granule proteins such as neutrophil elastase (NE) and myeloperoxidase (MPO) [2]. These large extracellular structures can trap and kill bacteria over a wide area. The extent to which NET formation occurs in specific clinical circumstances and the degree to which disorders caused by NETosis are clinically significant in acute illnesses, and the biological events that induce disorders of NETosis are generally unknown [4, 5].

Trauma predisposes to infection through mechanisms that are poorly understood, but cellular injury leads to release of immunologically active “damage molecules”, or DAMPs [6]. We found that these DAMPs include mitochondria (MT) and their remnants like mitochondrial DNA (mtDNA) [7]. MT evolved from saprophytic bacteria and became endosymbionts [8], so many molecular similarities still exist between bacterial DNA and mitochondrial DNA (mtDNA). We have already shown that purified mtDNA in the presence of PMN causes endothelial monolayers to become permeable for prolonged time [9]. Moreover, it is also known that NET release by PMN also contributes to endothelial activation [10, 11].

We therefore questioned whether mtDNA, due to its similarity to bacterial DNA, could act like a pathogen-associated molecular pattern (PAMP), and activate signaling pathways that lead to NET formation. Moreover, since our prior results shows that trauma leads to mtDNA release as well as predisposing to infection, we hypothesized that mtDNA released by injury might induce NETs in a dysfunctional manner leading to an increased likelihood of infection, non-specific inflammation or both. We therefore investigated the relationship between injury, circulating mtDNA and PMN NET formation.

## Materials and Methods

### Compliance

All studies were approved by the IRB at Beth Israel Deaconess Center. This included using peripheral blood for PMN preparation and discarded liver for mtDNA preparation. Written consent was obtained to draw blood. The IRB waved the need for the written consent to obtain mtDNA from discarded tissue.

### Preparation of mtDNA

Apparently normal human liver was obtained from the uninvolved margins of hepatic tumor resections performed at Beth Israel Deaconess Medical Center (BIDMC). MtDNA was isolated from these liver samples using the mtDNA Extractor CT Kit from WAKO Chemicals (Richmond, VA). The mtDNA was evaluated by quantitative PCR using mitochondria gene specific primers [7].

### Human PMN preparations

PMN were freshly isolated from blood for each experiment, as described earlier [12]. Our definitions for young and old in this manuscript are < 65 yo and ≥ 65 yo, respectively. Briefly, PMN were isolated from minimally heparinized whole blood using a one-step centrifugation procedure using PMN Isolation Medium (Thermo Fisher Scientific, Waltham, MA). The neutrophil layer was collected and osmolality was restored. Residual red blood cells were lysed briefly to increase PMN purity. Cells were washed and suspended in HEPES buffer [12].

### Detection of NETs

NETs were detected in fixed PMN using previously published methods [1, 13]. In brief, PMN (200,000–500,000 cells) were seeded on coverslips in 24-well plate. After stimulation cells were fixed with 4% paraformaldehyde and permeabilized with 0.05% Triton X-100. Cells were blocked with 5% donkey serum and then treated with a mixture of primary antibodies to neutrophil elastase (rabbit polyclonal, EMD Millipore, Billerica, MA) and histone H1 (mouse monoclonal, Santa Cruz Biotechnology, Dallas, TX). Then cells were treated with secondary antibodies, donkey anti-rabbit IgG-Alexa 488 and donkey anti-mouse IgG-Alexa 546 (both from Life Technologies, Grand Island, NY). Coverslips with cells were mounted with ProLong Gold anti-fade mounting media with DAPI (Life Technologies). NET formation was analyzed with a Plan-Apochromat 20×/0.8 lens using a Zeiss LSM 510 upright confocal microscope system. We performed a blind study. At least three images were taken randomly from different regions of each unidentified coverslip. Then the representative figures for each experimental condition were selected. Diphenyleneiodonium chloride (DPI; Sigma-Aldrich, St. Louis, MO) and suppressive oligodeoxynuclotide (ODN) TTAGGG (Invivogen, San Diego, CA) were used to block NADPH oxidase and TLR9, respectively.

## Results

### MtDNA induces NET formation in human PMN

**Fig 1:**
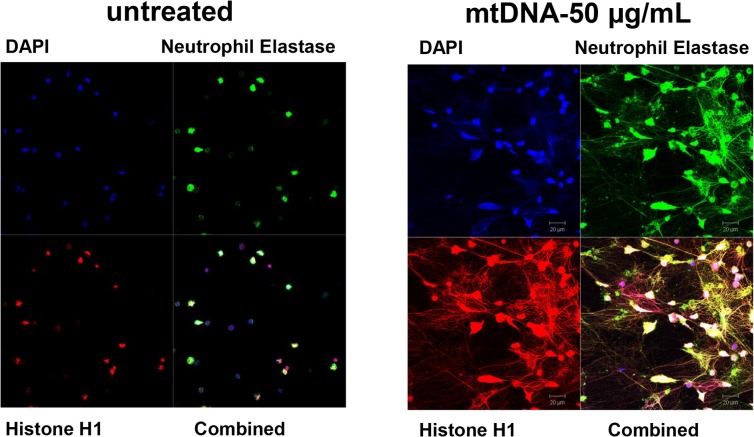
MtDNA induces NET formation. Freshly isolated PMN from young volunteers were attached to coverslips for 1 h and treated with either medium (untreated) or with purified mtDNA (50 μg/mL) for 4 h. NET formation was detected as described in Materials and Methods. Experiments were repeated more than three times. Magnification x40.

We first evaluated whether mtDNA induces NET formation in human PMN. As shown in **Fig. 1**, mtDNA (50 μg/mL) induced NETs in PMN freshly isolated from control subjects, compared to untreated cells.

### MtDNA-induced NET formation is independent of NADPH oxidase

**Fig 2:**
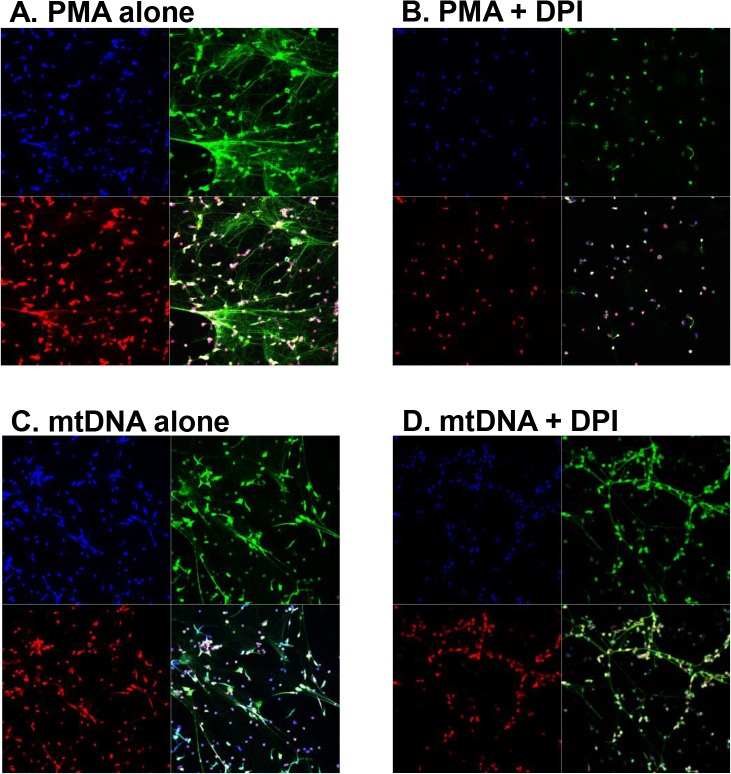
MtDNA-induced NET formation is independent of NADPH oxidase. PMN were pre-treated with 10 μM DPI for 1 h before the stimulation (B and D). Then either 20 nM PMA (A and B) or 50 μg/mL mtDNA (C and D) was applied for 3–4 h. NETs were detected as described in Materials and Methods. Experiments were repeated more than three times. Magnification x20.

The role of oxidants in NET generation depends on the specific stimulus [14]. It is well accepted that PMA-induced NET formation requires NADPH oxidase. We therefore evaluated the role of NADPH oxidase in mtDNA-induced NETosis. NET formation in response to 20 nM PMA for 4 h was completely inhibited in the presence of 10 μM DPI (**Fig. 2A and 2B**). In contrast, the induction of NETs by mtDNA (50 μg/mL) was not affected by DPI (**Fig. 2C and 2D**).

### MtDNA-induced NET signals via TLR9

**Fig 3:**
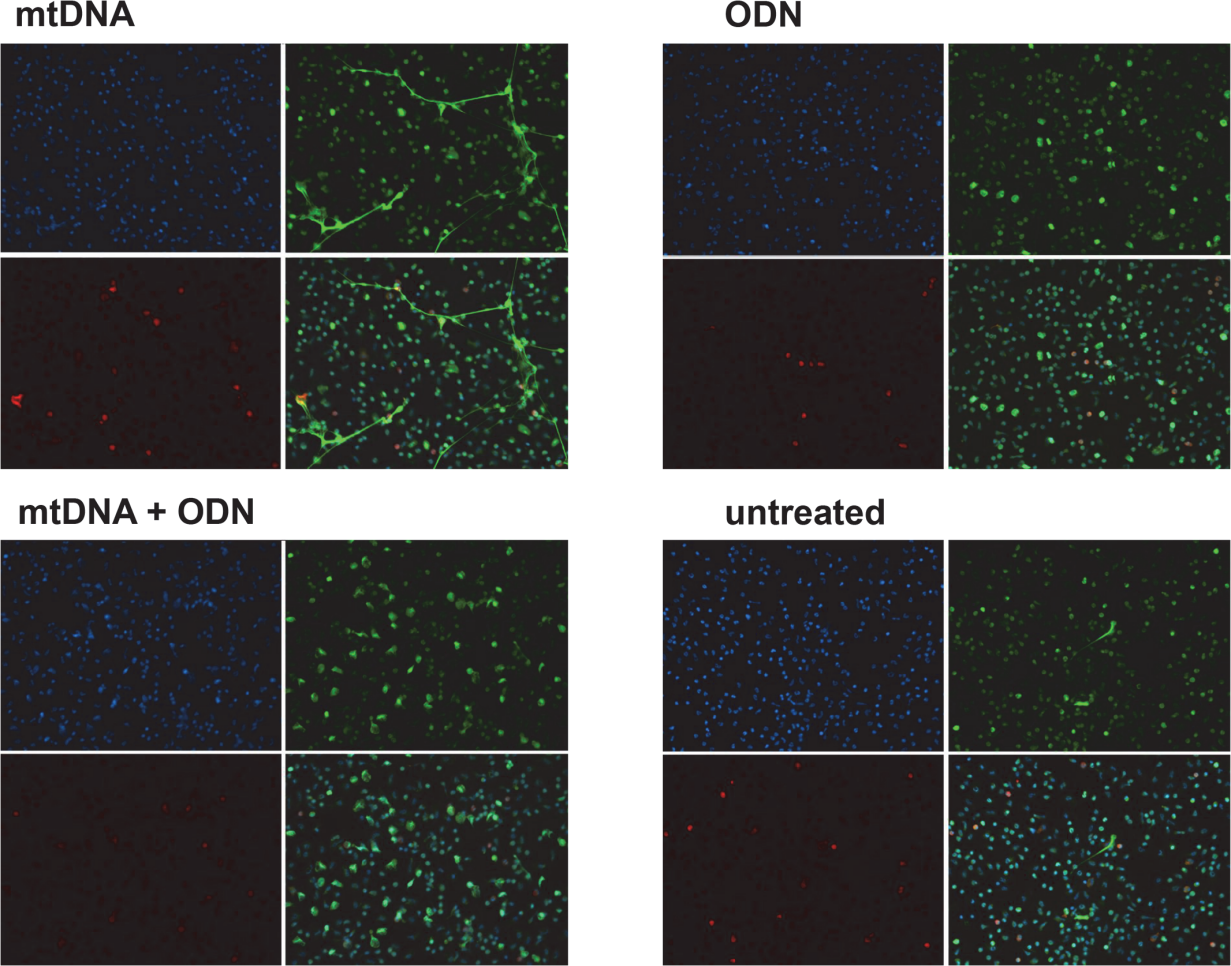
MtDNA induces NET formation via TLR9. PMN were attached to coverslips and treated with mtDNA (50 μg/mL, **A**), ODN (5 μM, **B**), mtDNA and ODN (**C**) or medium (**D**) for 3–4 h. NETs were detected as described in Materials and Methods. Experiments were repeated at least three times. Magnification x20.

We have shown that mtDNA-induced endothelial permeability increase was reduced in the presence of TLR9-specific inhibitor, ODN TTAGGG [9]. Thus we examined whether the ODN has an inhibitory effect on mtDNA-induced NET formation. MtDNA-induced NET formation was completely inhibited in the presence of 5 μM ODN (**Fig. 3**).

### Plasma levels of mtDNA in elderly are higher than in young people

**Fig 4:**
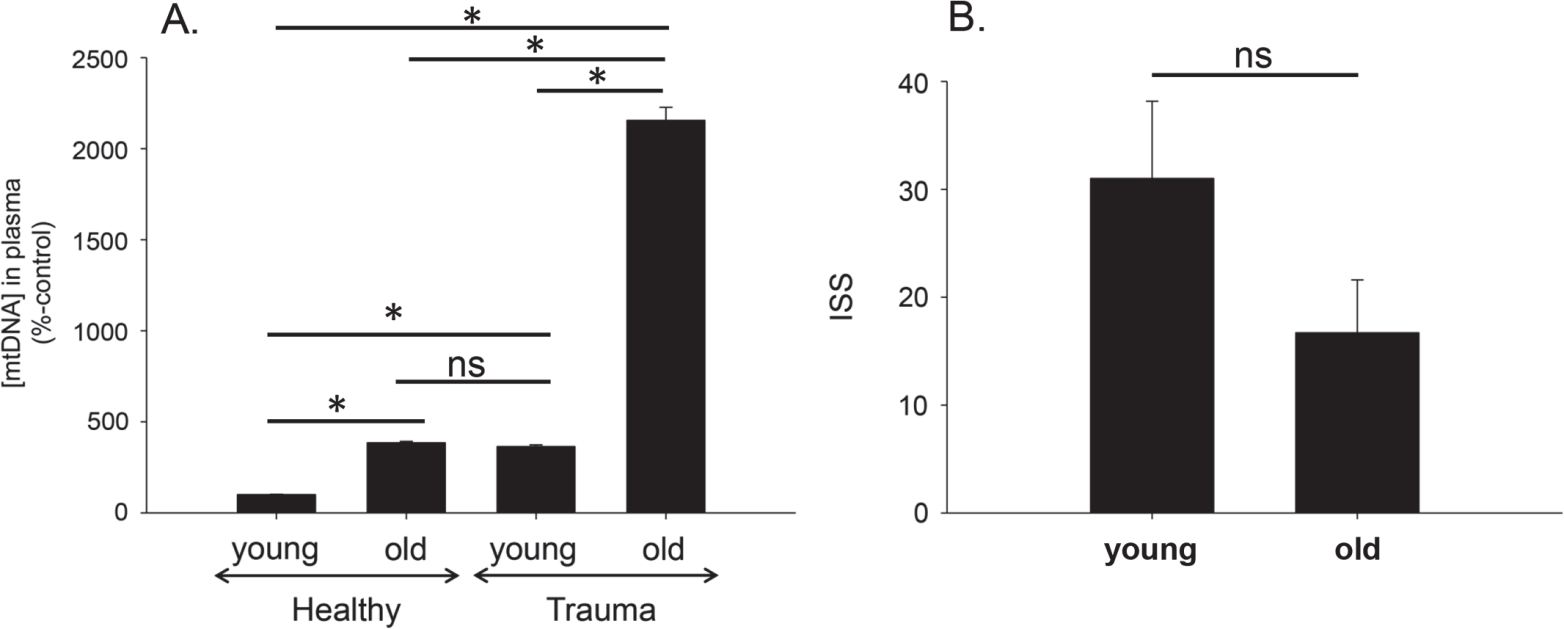
Plasma mtDNA levels are higher in the elderly. **A**. MtDNA concentration was assessed in plasma obtained from subjects from four groups: 1. Young healthy (n = 11, average age 23 years); 2. Elderly healthy (n = 7, average age 72 years); 3. Young trauma (n = 9, average age 37 years); and 4. Elderly trauma (n = 6, average age 83 years). All pairs showed significant difference except between old healthy vs. young trauma; p<0.001, One Way ANOVA, followed by Tukey’s Test. **B**. ISS is shown for young and older trauma patients listed in **A**. p = 0.164 (Student’s t-test).

We measured mtDNA content in plasma from young (n = 9, average age 36.8) and old (n = 6, average age 82.7) trauma patients and corresponding young (n = 11, average age ~25) and old (n = 7, average age 72.1) volunteers by quantitative RT-PCR using primers specific to the mitochondrial gene, cytochrome B. Assigning young volunteers’ mtDNA levels as a control value of 100%, elderly volunteers, young trauma patients and elderly trauma patients had values of 385 ± 8, 364 ± 9, and 2156 ± 72 respectively (**Fig. 4A**). All pairs were significantly different (p<0.001) except between healthy old and young trauma (n>0.05) by One Way ANOVA with Tukey’s test. Interestingly, the Injury Severity Score (ISS) was higher in young patients than elderly trauma patients; 31.0 versus 16.7, respectively (**Fig. 4B**). However, this difference was not significant (Student’s t-test, p = 0.164).

### NETs are induced in peripheral blood PMN from trauma patients

**Fig 5:**
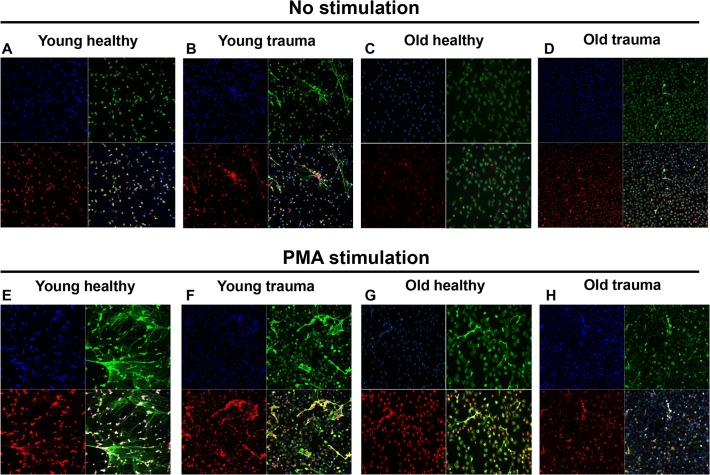
NET formation in young and old trauma patients and volunteers. NET formation in PMN isolated from young healthy volunteers (n = 10, **A and E**), young trauma patients (n = 7, **B and F**), old healthy volunteers (n = 6, **C and G**), and old trauma patients (n = 5, **D and H**) was evaluated. **A-D**: PMN were immediately fixed after attachment. **E-H**: PMN were stimulated with 20 nM PMA for 3–4 h.

We next evaluated whether PMN obtained from trauma patients might already be partially activated by their exposure to the circulating mtDNA released from injury sites. PMN from young and old volunteers did not show any NETs without *ex-vivo* stimulation (**Fig. 5A and 5C**). PMN from elderly trauma patients did not have many NETs (**Fig. 5D**) but PMN from young trauma patients showed a clear increase in NETs without any *ex-vivo* stimulation (**Fig. 5B**). Stimulation of young volunteers’ PMN with 20 nM PMA for 4 h induced a significant increase in NET formation (**Fig. 5E**). PMN from young trauma patients showed similar number of NETs after *ex-vivo* PMA stimulation as PMN from young controls (**Fig. 5F**). PMA induction of NETs was very limited in PMN from either healthy elderly volunteers or elderly trauma patients (**Fig. 5G and 5H**).

## Discussion

We explored the effects of mtDNA on NET generation in freshly isolated human PMNs, and looked for a mechanism of this activation. We also investigated whether aging modifies the PMN response to mtDNA. It is well established that NET formation is generally NADPH oxidase-dependent [15]. Indeed, PMA-induced NET formation was completely inhibited by NADPH oxidase inhibitor, DPI (Fig. 2). However, our *in vitro* data demonstrate for the first time that mtDNA stimulates human PMN to form NETs independently of NADPH oxidase via TLR9 (**Figs. 1–3**). Supporting our observation, others have shown that uric acid induces NET formation in NADPH oxidase-independent manner which involves activation of NF-κB [16].

Interestingly, healthy elderly and young trauma patients had similar plasma mtDNA concentrations (**Fig. 4A**). After trauma mtDNA levels were significantly higher in both the elderly and young but the elderly trauma group had significantly more mtDNA in their circulation than younger trauma patients even though their injury severity scores (ISS) were the same or if different, lower (p = 0.164; **Fig. 4**). It is completely unclear why this might be the case, but we might speculate that tissues in the elderly are less well perfused and therefore more at risk for necrosis after similar mechanical trauma. It might also be that mtDNA is less efficiently cleared in the elderly. But the effect is quite clear and may suggest a rationale for the clinical observation that elderly patients are more at risk for infectious morbidity and death after injury than younger patients. This observation of lower responses in the face of higher mtDNA levels suggests a form of immunologic ‘tolerance’ and requires further investigation. However, the evidence suggests that plasma mtDNA concentration might be a more precise way of estimating organ damage than traditional ISS in the elderly and hence a better predictor of exaggerated inflammation. The data could also suggest that increased plasma mtDNA after injury induces NETs that might predispose to bystander organ injury as in acute respiratory distress syndrome or multiple organ failure [7, 9, 17, 18].

It is not clear why PMN from elderly trauma patients have limited NET formation and we plan to investigate this further. We can speculate that PMN from elderly are less sensitive to mtDNA since TLR expression is attenuated in old mice [19]. There are, however, no published data concerning TLR expression in elderly humans.

Aging is associated with sub-optimal neutrophil responses, including migration and bactericidal activity [20, 21]. Considering the similarity between mtDNA and bacterial DNA we propose that PMN from elderly trauma patients might respond weakly bacterial as well as mitochondrial DNA. This could predispose to systemic bacterial infections like pneumonia, and it has been demonstrated that aged mice fail to form NETs in response to methicillin resistant *Staphylococcus aureus* (MRSA) infection [22]. NETs are not of course, the only PMN method of bacterial killing. Bacteria can be killed by phagocytosis with activation of NE [23] and reactive oxygen species [24]. Thus the elderly might also have limited bacterial killing ability due to reduced PMN enzymatic activity caused by aging. However, there is no published data that suggests that elderly people have reduced PMN enzymatic activity. Rather Sapey et al. showed that PMN from elderly people have increased granular release than PMN from young [25]. To our knowledge, data on age-related changes in bacteria-induced NET formation in humans have not been reported. We also observed that PMN from both healthy and injured elderly patients respond to PMA with much less NET formation than young adults (**Fig. 5G and 5H**). Taken together, these findings raise the possibility that decreased NET formation could contribute to the increased frequency of death seen from infection in elderly trauma patients. Indeed, recently published results suggest that elderly trauma patients die due to sepsis far more often than young patients [26]. Caution is required here, since although we detect NETs in PMN freshly isolated from blood of trauma patients, the real population of NET-forming PMN in the body is difficult to estimate. Some PMN might be cleared after NET generation *in vivo* and/or some of NETs could be trapped in various organs/body tissues. Therefore, the NETs we detected without any further *ex-vivo* stimulation might represent a very limited number of total NET-forming PMN.

Venous thromboembolism (VTE) is also a common event in surgical patients that may lead to fatal pulmonary embolism (PE) [4, 5]. Fuchs et al. found that NETs caused platelet adhesion, activation, and aggregation when perfused with blood [27] and that NETs induced thrombus formation and coagulation. NETs may also play a role in animal models of deep vein thrombosis (DVT) [28]. The mechanisms of DVT and PE after injury are still largely unclear, but our data suggest that increased circulating mtDNA after injury might be involved by virtue of inducing NETs that favor thrombus formation. It is well known that the risk of DVT increases with age [4, 5] and that DVT is multifactorial. But our findings suggest the possibility NET formation and mtDNA might play a role.

In summary, our data demonstrate that mtDNA induces NETs through a TLR9 dependent and NADPH oxidase-independent pathway, and that elderly trauma patients generate fewer NETs than young patients despite equivalent or lesser injury and higher circulating mtDNA. Further study of the links between circulating mtDNA and NET formation may therefore help elucidate the mechanisms of trauma-related susceptibility to secondary bacterial infection, thrombosis, and organ failure, especially in the elderly.
